# Geometry based gene-expression signatures detect cancer treatment responders in clinical trials

**DOI:** 10.1186/s12885-026-17312-4

**Published:** 2026-11-10

**Authors:** Wojciech Chachólski, Ryan Ramanujam

**Affiliations:** 1 Department of Mathematics, KTH-Royal Institute of Technology, Stockholm, 10044 Sweden; 2 Department of Clinical Neuroscience, Karolinska Institutet, Stockholm, 17176 Sweden

**Keywords:** Gene-expression, Cancer, Topological data analysis

## Abstract

**Background:**

Topological data analysis has had many developments with potential application to precision oncology. The goal of this study is to determine if using geometry-based methods of gene-expression signatures of tumors can be used to both create a definitive classification of responders and non-responders, and to predict patient treatment response using out-of-sample testing methodology.

**Methods:**

Gene-expression signatures of tumors, constructed from geometrical attributes of data, were created and tested in an open-sourced Pfizer clinical trial on avelumab plus axitinib in advanced renal cell carcinoma. This was a previously conducted and published Phase III randomized controlled trial as part of the JAVELIN Renal 101 project (2019). Multiple geometries of different properties based on data organization were made using gene-expression data (*n* = 726), which were then used to compile aggregate synthetic subgroups of response for every patient as an internal classification. Additional and separate out-of-sample testing of patients was conducted, using the remaining samples to build a geometrical scaffolding of response, and then creating a similar synthetic grouping then tested via the log-rank test. Results were compared against three published gene-expression signatures, one derived from this cohort and two externally.

**Results:**

Geometrical gene-expression signatures were successfully used to create internal classification of responders to the intervention, with dramatically different Kaplan-Meier (KM) estimators based on responder assignment. Furthermore, out-of-sample prediction based on leave-one-out methodology was able to correctly predict this responder classification with 82.9% accuracy at median split of response, with area under the curve of 0.880 (95%CI 0.855–0.905). Gene-level analysis showed the signal to be a co-expressed program rather than individual transcripts. No gene retained an independent association after conditioning on its co-expressed neighbors, and two to five co-expressed modules exhausted transcriptomic information about classification (out-of-fold AUC 0.74–0.76), exceeding penalized regression on all 22,955 genes (0.73). Adjusted restricted mean survival time at 14 months was + 2.54 months in predicted responders versus + 0.44 in non-responders, an adjusted interaction of + 2.10 months (*p* = 0.004), which predicted responders accounting for 85.3% of the between-arm RMST gain. In unadjusted models, none of the published signatures reached a significant interaction (ratio of hazard ratios 0.67–0.82, all *p* > 0.06), against 0.55 (*p* = 0.005) for our classifier. Covariate adjusted Cox and RMST regression models gave consistent estimates at cutpoints below 10^− 3^, and 20,000 permutations tested supported the continuous interaction (*p* = 0.019; family-wise adjusted *p* = 0.042).

**Conclusions:**

Gene-expression based geometrical signatures can be used to create an internal reference classification of responders and non-responders in clinical trial data and are also highly accurate at predicting these labels out-of-sample, detecting treatment effect heterogeneity that established signatures did not. These methods can be used to identify target biomarkers of response, which may support enrichment or stratification designs.

## Introduction

Precision medicine is individualizing the treatment for a patient based on characteristics such as demographics and clinical information, or experimental data such as genomics, gene-expression, proteomics, etc. By considering individual response, more efficacious treatments can be selected [1].

Mutations are the most widely used biomarker for dividing patients and tumors into clinical subgroups [2], typically a single somatic mutation or a combination of several. This has led to efforts to find biomarkers across tumor types which may indicate improved treatment response [3]. The ability to detect individuals who may respond to a treatment across various tumor tissues would have strong clinical utility for precision oncology [4]. Nevertheless, identifying such biomarkers to detect a large proportion of patients with efficacious responses remains difficult [1].

Some cancer therapies have been FDA approved based on the presence of certain mutations [5]. When examining small molecules, tumor viability screening data from the Dependency Map project (DepMap) [6] presented evidence that gene-expression is the data type most strongly associated with such drug sensitivity in cell-lines. Given this, efforts to combine gene-expression data into signatures which correspond to tumor biology and/or state are warranted for drugs with direct tumor modulatory activity. One method of combining complex data, such as RNA-seq, is by using techniques in geometrical and topological analysis.

Topological data analysis (TDA) is a rapidly growing field which structures and organizes data based on geometry. It transforms data into invariants that capture higher order interactions and are amenable for statistical and machine learning analysis. The presence of higher order information can often yield insights which are not available with standard statistical methods. This field has been applied to biomedical research, with results in breast cancer characterizing tumors with unique characteristics [7]. It also was used to identify Type 2 diabetes (T2D) subgroups based on clinical features which had distinct genetic associations [8].

We have developed several TDA methods based on classical TDA approaches such as extending the mapper algorithm for multi-measurements [9], and creating a new invariant called stable rank [10, 11] which is a key component of the technology presented here. Stable rank has also been used to create supervised learning methods [12], which have been applied to characterization of sex-based differences in microglia [13]. Extensions to these methods herein create signatures based on gene-expression, which contain various unique properties and can be correlated to outcomes of interest such as treatment response.

Testing these methods to detect biomarkers of response is desirable in an oncology randomized controlled trial. One such available trial was conducted by Pfizer for avelumab plus axitinib in patients with advanced renal cell carcinoma as part of the JAVELIN Renal 101 program. The Phase III trial reached clinical endpoints, and the drug combination was approved. The data from this trial was released publicly, and included information on patient clinical features and outcomes as well as high-throughput data such as gene-expression, mutation, HLA, etc. The release of this data provides an ideal test case for using geometrical methods to predict treatment response in an oncology clinical trial.

The objectives of this study are to create gene-expression signatures, to create and apply a new methodology to the Pfizer clinical trial to determine if subgroups of responders can be identified based on these signatures, and to determine if unbiased prediction of sample responses to the intervention can be made with high accuracy.

## Materials and methods

### Pfizer JAVELIN Renal 101 trial

The Pfizer JAVELIN program consisted of long-term monitoring of patients with advanced renal cell carcinoma [14]. A Phase III trial of avelumab plus axitinib was conducted with a comparator arm of sunitinib as the standard of care. The total number of patients enrolled was 886, with 442 assigned to the treatment arm and 444 assigned to the sunitinib comparator arm. The primary endpoint was progression-free survival (PFS).

Data for this trial was open sourced by Pfizer; of the data provided, 726 patients included gene-expression data, along with clinical parameters and outcomes. Gene-expression was given in the form of log_2_ TPM, i.e. Transcripts Per Million, with minimum values set to 0.01. Expression values were used as provided by the trial sponsor, in TPM, without gene filtering or imputation. The matrix was complete with no missing values at any stage. Sample quality was compared between randomized arms using four per-sample proxies derived from expression alone: the fraction of transcripts at the detection floor, median non-floor expression, ribosomal transcript fraction, and expression entropy. Groups were compared by Welch’s t-test and by standardized mean difference. Mitochondrial transcripts, the most standard degradation proxy, are absent from the released expression matrix and could not be assessed.

Clinical information included baseline parameters such as age, PD-L1 positive status (PD-L1+), sex, time to PFS, and binary indication of censoring at PFS. In the full trial population, the median time to PFS was reported as 13.3 months in the treatment arm and 8.0 in the comparator arm, giving a stratified HR of 0.69 (*p* < 0.0001). Values reported in this paper differ slightly, as they are derived from the 726 patient subset with gene-expression data and clinical outcomes, rather than all 886 randomized patients.

### TDA approach

The overall approach of this paper is to extend our existing methodology, described below, for constructing collections of subgroups organized into covers. These covers are indexed by sets of parameters, which we refer to as geometries. A geometry specifies the parameters that govern the transformation of data points in a metric space into functions, which are subsequently aggregated for downstream analysis. In this paper we build upon the existing framework by introducing two related concepts. First, for each data point in a dataset, we construct synthetic groupings that are used for internal classification. Second, we introduce a leave-one-out prediction procedure in which geometries are constructed from the dataset with the target sample excluded, and the resulting structures are then used to predict the omitted sample.

Beginning with the existing method, we build from the local approach as previously described [15]. This process assigns, to every element in a distance space, a non-increasing function called a stable rank according to some predefined parameters. A key feature of this signature of a point in a distance space is that it captures some geometrical properties of the embedding of this point into the entire distance space. In particular, points whose embeddings have similar geometrical properties have similar stable ranks, and the embeddings of points whose stable ranks are far apart have different geometrical properties.

The first step of our analysis is to choose a collection of sets of parameters (called geometrical parameters) for extracting stable ranks. There are two kinds of parameters, one related to homology, and one related to the geometry determined by distances. Geometrical properties are encoded by specifying distributions characterizing various regions of the distance space. The parameters used are defaults for this process, and include number of samples to average (1000), number of points sampled (30), distributions used (normal with variance [0,1]), overlap and step size (25% and 15% of the data, respectively) and filter function (l2 distance on the stable ranks).

Extracting stable ranks, with respect to each set of geometrical parameters, is the second step of our analysis. In the third step, for every set of geometrical parameters, we consider the distribution of integrals of the obtained stable ranks. We think about these integrals as a filter function and apply local clustering according to this filter and the *l*_2_ distance in a process similar to the mapper procedure [7]. In this way, for every set of geometrical parameters, we construct various subgroups of the obtained stable ranks, and hence subgroups of the points in the distance space from which we started.

Thus the points in the distance space are grouped according to the geometrical properties of their embeddings as captured by the obtained stable ranks: the embeddings of the points in different subgroups have different geometrical character and the embeddings of the points in the same subgroup have similar geometrical character.

**Fig. 1:**
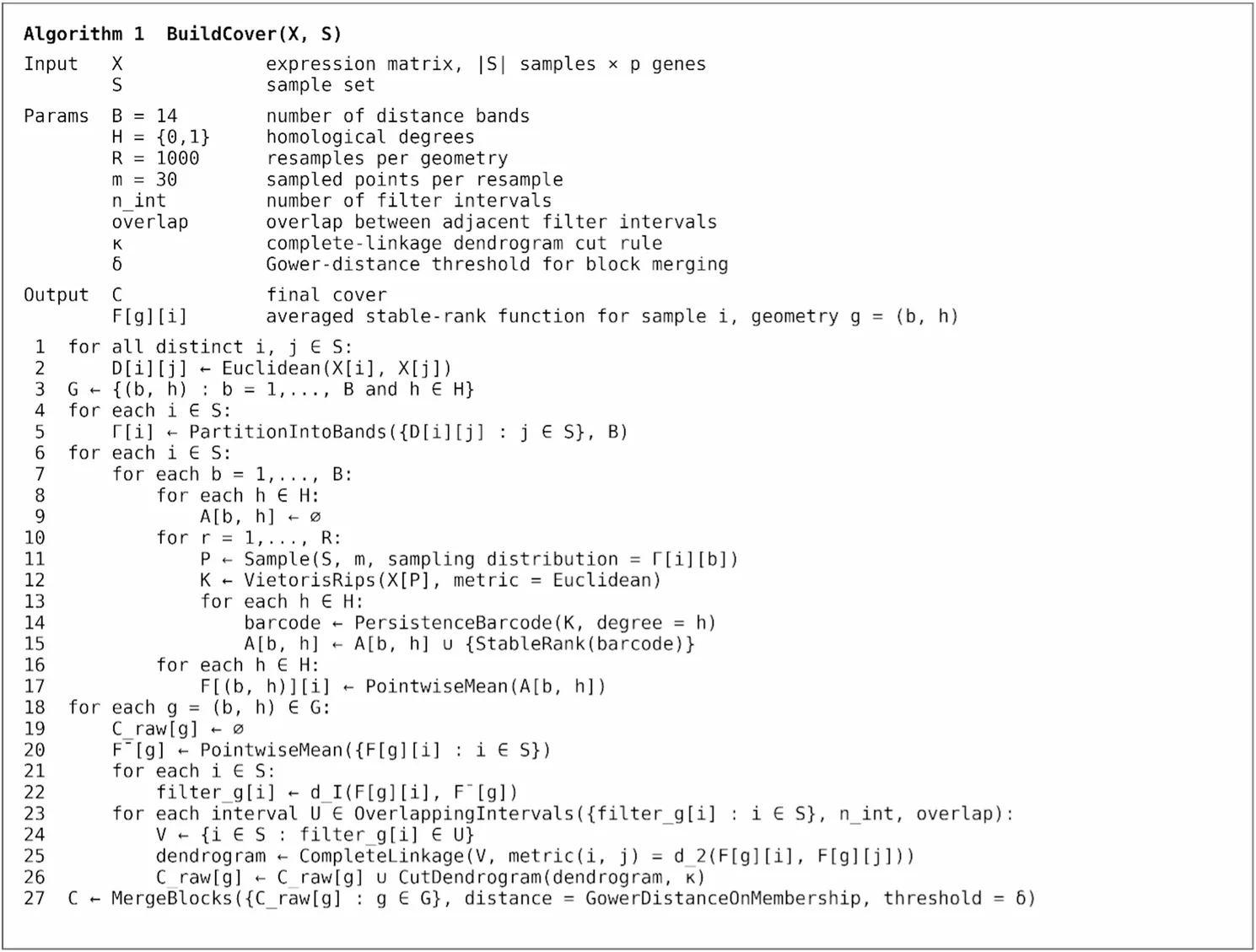
Pseudocode of the cover generation procedure, in generic form.

We applied this three-step process to the gene-expression data with the distance given by the Euclidean metric and 14 standard sets, or bands, of geometrical parameters in each of homological degrees 0 and 1. The outcome is a collection of subgroupings of patients, also referred to as blocks, based on geometrical similarities of the embeddings of the associated gene-expression in the Euclidean distance space given by the entire gene-expression data. These subgroups are organized into 28 groups, corresponding to our choice of sets of geometrical parameters, and these groups are referred to as geometries. See Fig. 1 for the pseudocode of this procedure, called Algorithm 1.

### Internal responder classification

The first new concept and a key innovation of this paper is a method to determine the internal classification of individuals, or samples, as a responder or non-responder. The method is applicable to all patients, regardless of censoring or outcome, so that no classifications are chosen based on i.e. reaching the event or censoring at an earlier or later time.

The procedure is conducted in the following iterative manner for every sample in the dataset. First, subgroups are formed based on the Euclidean geometry of the expression data across all the sets of chosen geometrical parameters as described in 2.2. Next, for each sample, all the subgroups containing it are combined into a synthetic block by taking the union of all samples contained within, with repeats. The aggregate of the synthetic block’s Kaplan-Meier estimator was constructed, and a log-rank statistic computed comparing treatment arms within it. This metric serves as a continuous score ranking each sample by the strength of treatment separation across its geometries, and is referred to as the internal reference score. Because the block is an aggregate in which a patient will recur, this quantity does not follow the null distribution of a log-rank p-value computed from independent observations.

This score is used directly when applicable, such as gene level characterizations. For some applications, a cutpoint is used to create categorical comparison. This cutpoint is prespecified as a median split, which falls at 2 × 10^-5^ and gives 364 reference responders and 362 non-responders. Every patient in the trial receives such a classification for internal reference response, including those in the comparator arm and those censored, regardless of time.

**Fig. 2:**
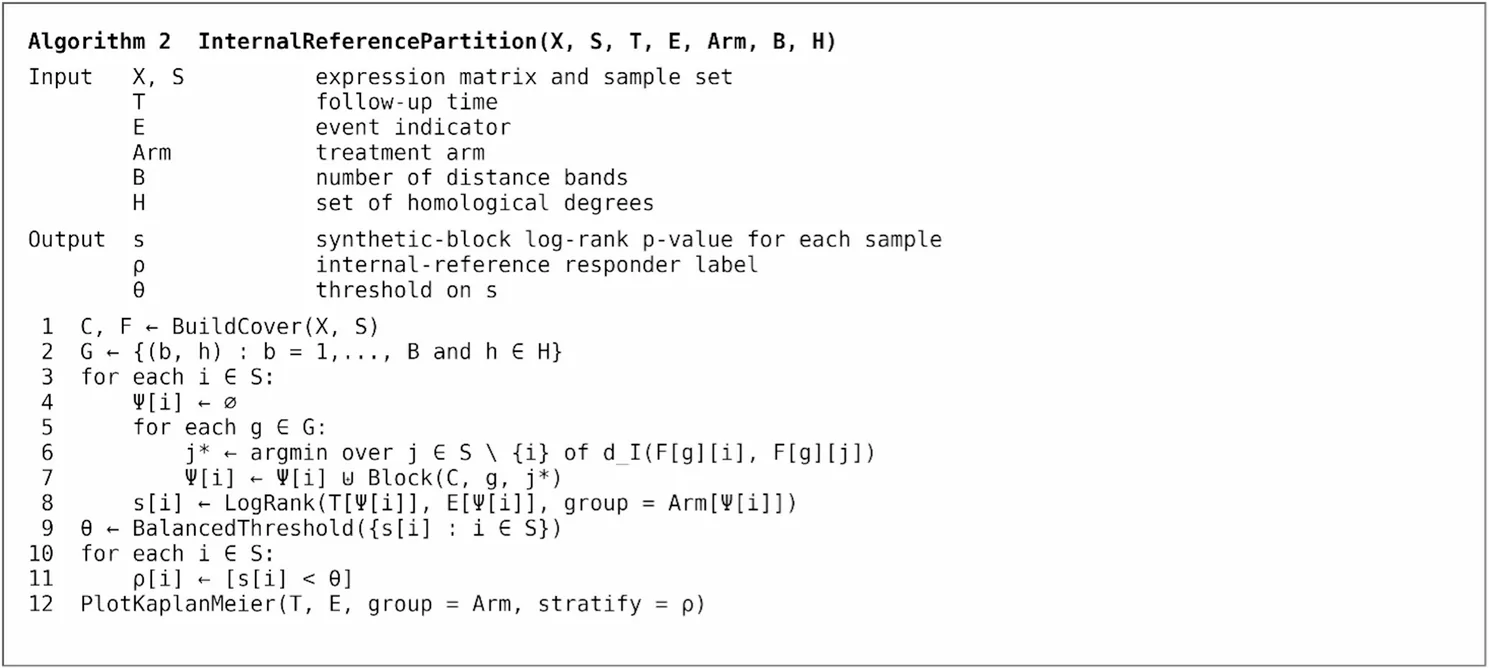
Pseudocode of the creation of internal responder reference classification, by creation of synthetic blocks for each sample, within the data.

This method is not designed to be unbiased, but rather to give internal reference classification of responders and non-responders. This represents an idealistic partitioning of response classification since it utilizes thresholding of synthetic blocks created via the sample’s membership. Figure 2 gives the pseudocode of Algorithm 2 for building the internal reference partition.

### Responder testing (leave-one-out)

To determine the utility of these methods to predict patients according to response, leave-one-out (LOO) cross-validation was conducted. The purpose was two-fold: to compare out-of-sample prediction with the response classification, and to measure the treatment effect captured by cross-validated predictions. Each sample was removed and the procedure as stated was applied to form subgroups for the rest of the cohort (725 individuals), to create a baseline for comparison. This ensures that the entire process was conducted without the sample test point included to ensure no contribution of the sample even for the geometry construction.

**Fig. 3:**
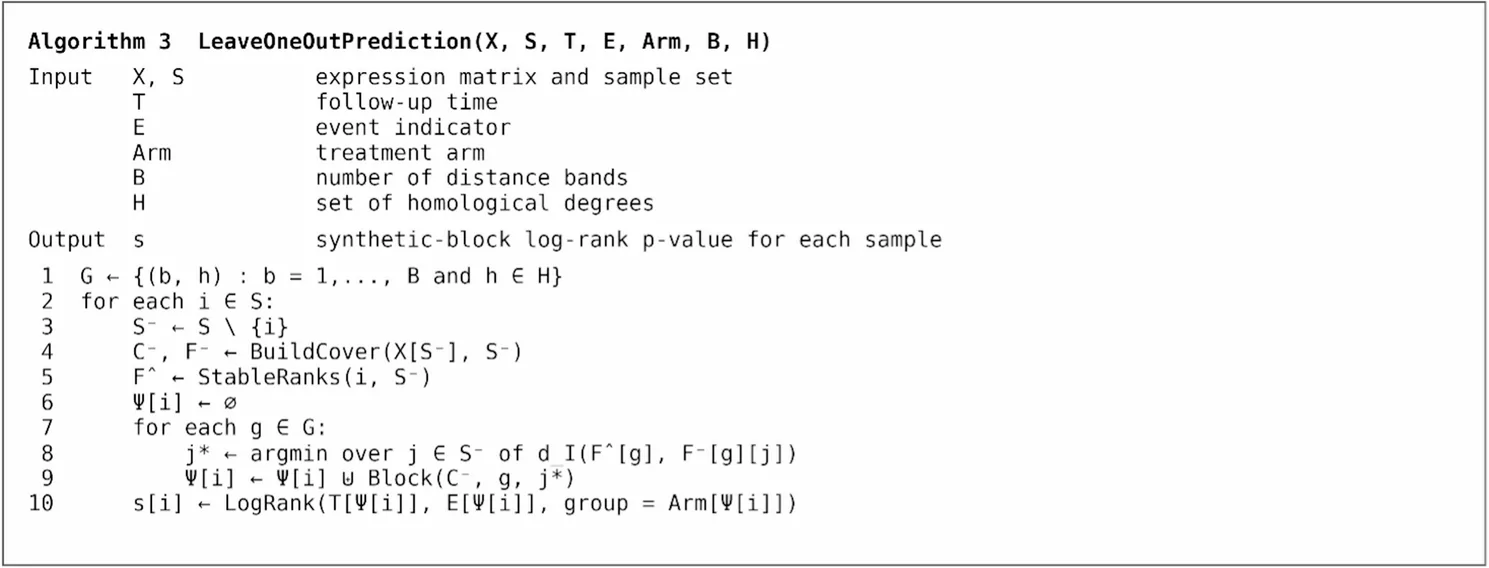
Pseudocode of the leave-one-out testing procedure, by creation of synthetic blocks for each sample with the sample completely removed from the geometries, and then subjected to some threshold for class prediction.

The test procedure continued by creating a synthetic block for the testing sample in the following manner. First, using the same procedure as for classification, the geometrical representations of the training data (*n* = 725) were created. Then, the excluded testing sample was transformed into stable ranks. Next, for each set of geometrical parameters one sample from the 725 individuals was chosen whose corresponding stable rank was the nearest (in *l*_2_ distance) to the stable rank of the testing sample. A synthetic block was then formed as the union of the blocks of these nearest chosen samples across all the sets of geometrical parameters. After this procedure was completed, the KM of the synthetic block was tested using the log-rank test, and the p-value was recorded as the out-of-sample prediction score for that patient. Similar to the internal reference, this prediction score is not tested directly for statistical significance. This was repeated for all samples, giving out-of-sample measurements for every patient with log-rank p-values for categorization. The pseudocode for this procedure is presented in Fig. 3.

Kaplan-Meier plots were made at response groupings with various thresholds of p-values (5 × 10^-2^, 1 × 10^-2^, 1 × 10^-3^, 1 × 10^-4^, 5 × 10^-5^, 1 × 10^-5^), based on the computed log-rank tests of each patient as calculated out-of-sample. Hazard ratios were estimated by Cox proportional-hazards regression, with the proportional-hazards assumption assessed by Schoenfeld residuals. Restricted mean survival time (RMST) was estimated at τ = 14 months, a value within the observed follow-up for both arms [16]. Models fitted unadjusted and were then adjusted using age, sex and PD-L1 status.

Treatment-effect heterogeneity was assessed by the treatment × predicted response interaction, which for Cox models is the ratio of hazard ratios. For RMST models, the difference in treatment effect between groups was used, as estimated by pseudo-observation regression with generalized estimating equations and robust standard errors [17].

The internal reference score was dichotomized at its own median, 2 × 10⁻⁵, giving 364 reference responders and 362 non-responders. This defined the labels against which predictions were scored. The predicted score was also dichotomized at its own median, 5 × 10⁻⁵, giving 362 predicted responders and 364 predicted non-responders; this then defined the prediction class. The area under the curve requires only the first, since it is computed across all utilized cutpoints of the predicted score, whereas sensitivity, specificity, predictive values and κ require both.

Predicted scores were compared with the internal reference classification across the full range of possible cutpoints. Discrimination was summarized by the area under the curve (AUC) of the receiver operating characteristic (ROC) with a DeLong confidence interval [18], computed with the pROC package [19]. Agreement at a given cutpoint was defined as concordance of out-of-sample prediction p-value with that of the rest of the cohort (*n* = 725), with respect to the cutpoint, and was summarized by Cohen’s κ with a normal-approximation interval [20] and interpreted against Landis–Koch bands [21]. For display and for the survival analyses that require a dichotomy, predicted scores were split at their median, 5 × 10⁻⁵. All principal analyses treated the score as continuous.

It is important to note that the internal response reference classification methodology and response prediction were independent procedures.

### Gene level characterization

All 22,955 genes were ranked against the internal reference score by Spearman correlation with Benjamini–Hochberg control of the false discovery rate, using the score directly [22]. No genes were filtered and no subset was pre-selected. Four hundred and seventy-five genes with zero variance across the cohort were excluded, leaving 22,480. Scores were reported to five decimal places, so the 333 patients (46%) whose score fell below 1 × 10⁻⁵ were ​recorded as zero and shared a single tied rank; the gene ranking was therefore determined by the ordering of the remaining 393.

Because strongly co-expressed genes cannot be reliably selected, for each top gene we counted how many others correlated with it at |r| ≥ 0.7 and ≥ 0.5 across the cohort, using raw Pearson correlations of log2 TPM, and retested each gene after subtracting the sign-aligned mean expression of the genes correlating with it at |r| ≥ 0.5. The 2,000 most strongly associated genes were then clustered by average linkage on 1 − |r| cut at a distance of 0.65, each cluster of five or more genes summarized by its first principal component, and partial correlations recomputed after conditioning on the leading clusters in turn [23].

Reproducibility was assessed by drawing 200 subsamples of 80% of patients, re-ranking all genes in each, and recording the overlap of the top 100 with the full-cohort ranking and between subsample pairs. The same protocol was applied to a median split of the score, to dichotomies of observed survival time, and to the union of the leading cluster memberships.

We next investigated how much of each geometric classification is recoverable from expression alone, using two targets: the internal reference classification and the leave-one-out prediction. Within each training fold, genes were ranked against the relevant continuous score and clustered as above. For evaluation, each target score was dichotomized at its own median since the area under the ROC curve requires a binary label. Four predictors, the two and five leading cluster eigengenes, the sign-weighted mean of the top 100 genes, and L2-penalized logistic regression on all genes were fitted on each training fold of a 10-fold cross-validation. These were then evaluated on held-out patients, with ranking, clustering, eigengene construction and model fitting all performed inside the fold. Discrimination was summarized as the area under the ROC curve on the pooled out-of-fold predictions.

To establish whether the modules also recover the treatment-effect heterogeneity of the full classifier, out-of-fold module scores were tested for a treatment × score interaction by Cox regression adjusted for age, sex and PD-L1, with the treatment effect within each half of a median split summarized as a difference in restricted mean survival time at τ = 14 months, unadjusted. Finally, a reduced panel was constructed as the mean of the k/2 most positively correlated genes minus the mean of the k/2 most negatively correlated and evaluated out of fold at sizes from 5 to 500 genes.

### Permutation testing

Permutation testing was applied only to leave-one-out predictions. The internal reference partition is constructed by creating synthetic blocks with strong treatment separation and is thus presented as an idealized construct, with no claims of inference and hence no permutation testing.

Treatment labels were permuted within strata defined by PD-L1 status and predicted responder group. Each patient row retained the survival time, censoring status, covariates and predicted score, so that only the arm label was shuffled and both censoring and expression data were preserved. This requires the predicted score to be independent of the patient’s treatment arm, which was verified (Wilcoxon *p* = 0.745; responder proportions by arm 52.2% versus 52.5%, χ² *p* = 0.98). Twenty thousand permutations were drawn, and all analyses below were computed from the same draws.

The test statistic was the z-statistic of the treatment × predicted response interaction from a Cox proportional-hazards model adjusted for age, sex and PD-L1 status. In the primary analysis, the predicted score entered as a continuous rank-transformed covariate, so no cutpoint was required. As a supplementary analysis, the same statistic was computed at five points on the predicted score; family-wise error across these was controlled by the Westfall-Young min-p permutation procedure [24], which accounts for the correlation among nested subsets, and the false discovery rate was controlled using the Benjamini–Hochberg procedure.

To assess how often a partition of this kind arises by chance, 20,000 random partitions matching the observed group sizes were generated and the interaction was recomputed in each.

The classifier itself was not re-derived under permutation, as rebuilding the geometries and cover for each permuted dataset requires approximately 10 days per run. The permutation null is therefore conditional on the observed predicted scores. Analyses used R 4.4.1 and the survival package.

### Comparator gene-expression signatures

Three published gene-expression signatures were evaluated on the same cohort and endpoint: the 26-gene JAVELIN Renal 101 Immuno signature [14], the IMmotion150 Teff signature [25], and the 18-gene tumor inflammation signature [26]. Signature scores were computed as the mean of z-scored log2 expression across the constituent genes, with standardization parameters and the median threshold derived from the training set under the same leave-one-out structure used for our own classification. Signature directions were fixed a priori from the source publications, and all constituent genes were present in the expression matrix by exact symbol match.

The Renal 101 Immuno signature is additionally reported under median aggregation, the form used in subsequent reanalyses of this cohort. Where a source publication specified weights that are not publicly available, the signature was scored unweighted. Each signature was split at the median of its own score and passed through the same Cox and restricted mean survival time interaction models applied to our classification, without covariate adjustment, since no covariate specification is defined for these signatures in their publications. Our reimplementation of the Renal 101 Immuno signature reproduced the published within-arm hazard ratios (0.62 versus 0.60 in the combination arm; 0.93 versus 0.89 in the control arm).

## Results

**Fig. 4:**
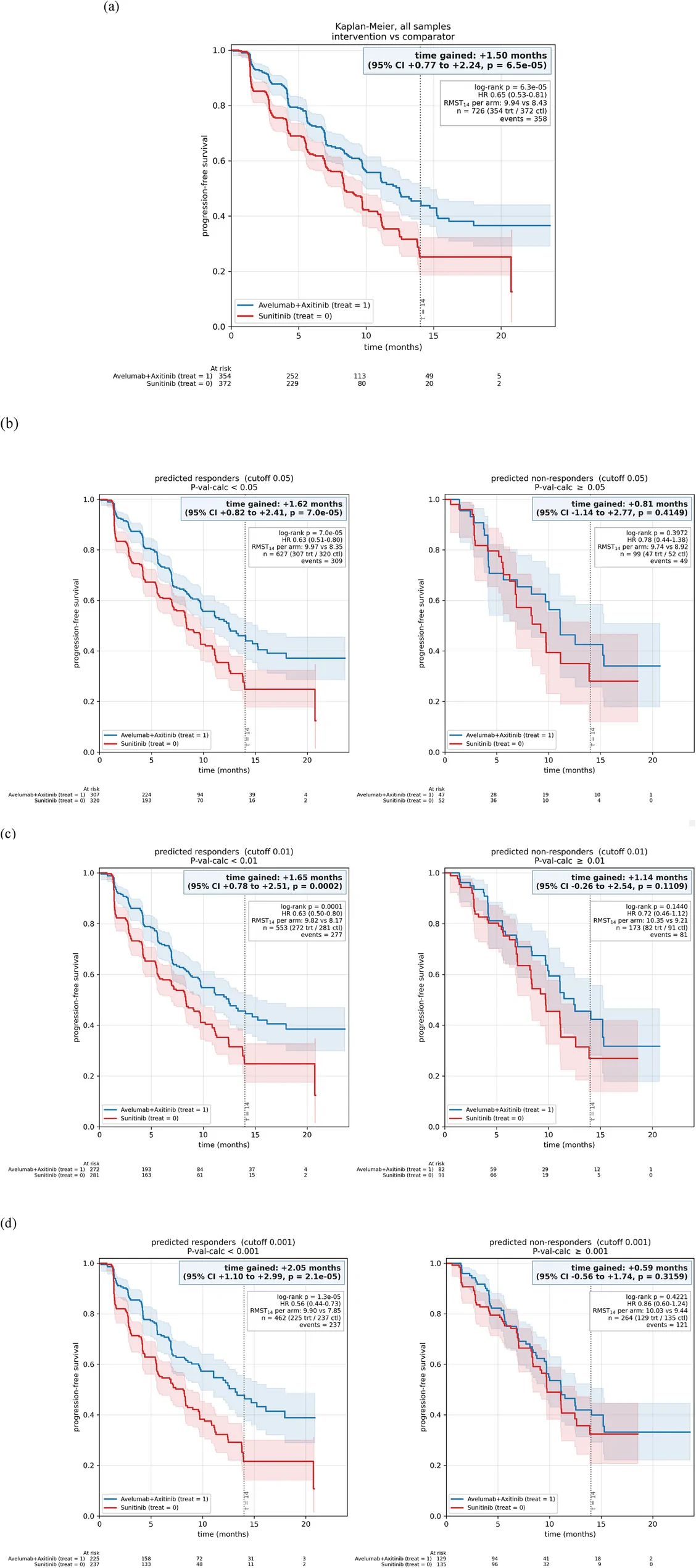

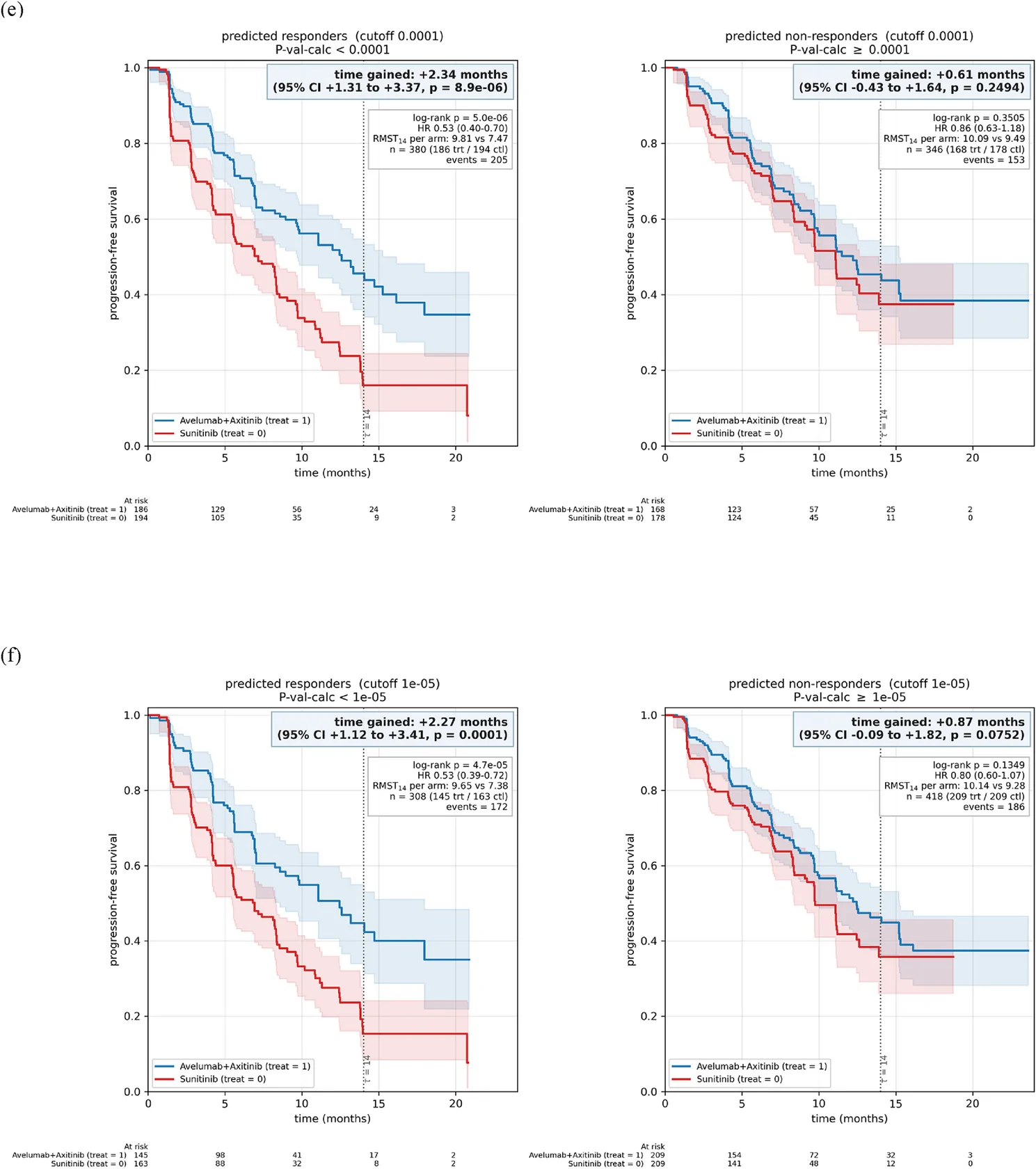
Kaplan-Meier plots of (**a**) all patients from the original study for which gene expression data was available (*n* = 726), and then out-of-sample predictions of responders (left) and non-responders (right) for p-value cutoffs of (**b**) 5 × 10^-2^, **c** 1 × 10^− 2^, **d** 1 × 10^− 3^, **e** 1 × 10^− 4^, **f** 1 × 10^− 5^.

Baseline characteristics for the cohorts are given in Table 1. Age, sex, and PD-L1 status were balanced across arms as expected due to randomization. There were 157 events in the avelumab plus axitinib arm compared with 201 in the sunitinib arm (*p* = 0.011). Median survival was 12.5 months versus 8.4 months, hazard ratio 0.65 (95% CI 0.53–0.81), log-rank *p* = 6.3 × 10⁻⁵ (Fig. 4a).

**Table 1:** Baseline characteristics by treatment arm.

Characteristic	Sunitinib (*n* = 372)	Avelumab+Axitinib (*n* = 354)	*p*-value
Age, years - mean (SD)	60.3 (10.1)	61.1 (9.9)	0.251
Female - n (%)	81 (21.8%)	97 (27.4%)	0.094
PD-L1 positive - n (%)	271 (72.8%)	245 (69.2%)	0.318
TCGA cluster m3 - n (%)	130 (35.0%)	101 (28.7%)	0.006
PFS event observed - n (%)	201 (54.0%)	157 (44.4%)	0.011

Description of trial arms by demographic characteristics, n(%) and p-value across groups

Expression was analyzed without filtering. No sample-quality proxy differed by randomized arm (all *p* > 0.06, largest standardized mean difference 0.14), giving no evidence of technical imbalance between treatment arms.

### Internal reference classification of response

**Fig. 5:**
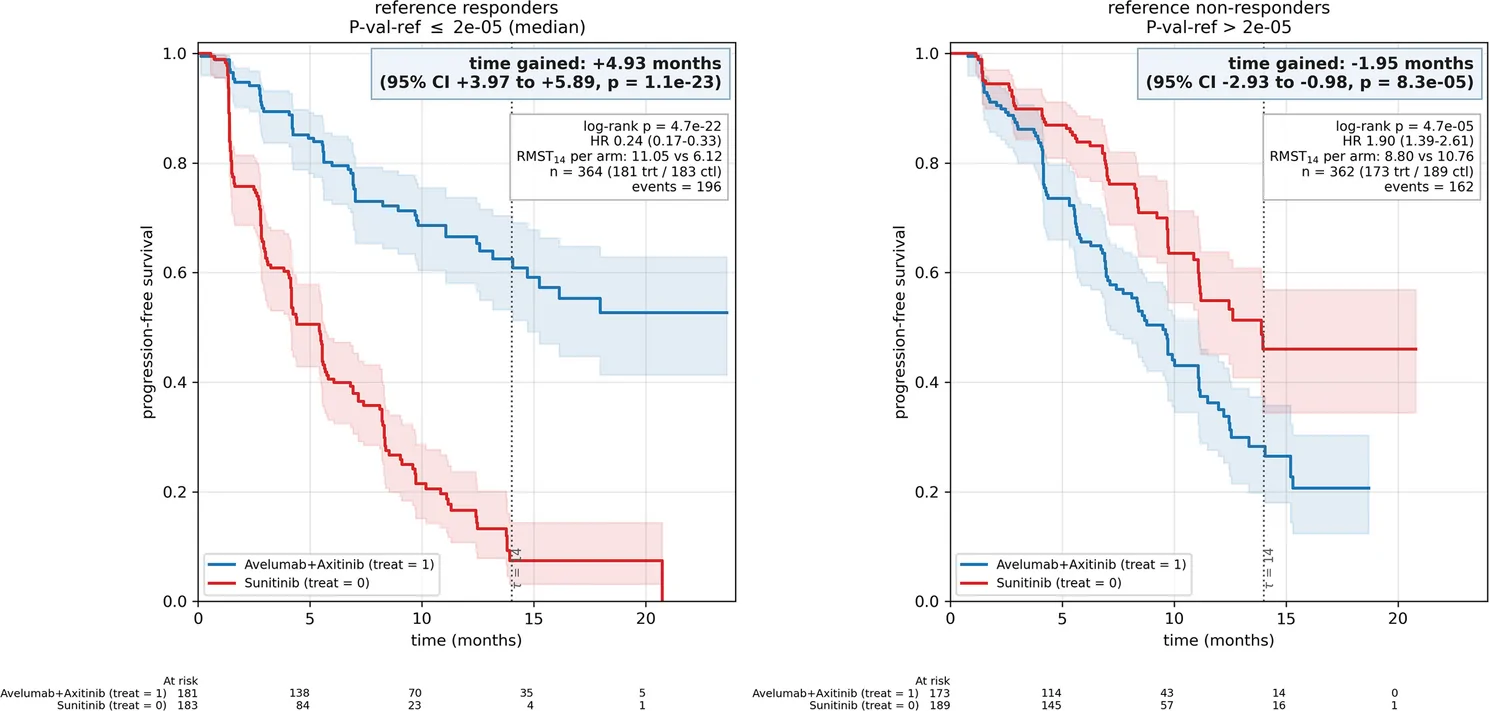
Kaplan-Meier plot of internal reference classification of response, by responder label. The reversal of effect direction is a consequence of intentionally partitioning for maximal response differences.

The procedure of creating synthetic subgroups per patient, using the internal data, was conducted and log-rank p-values computed. The dataset was then dichotomized at the median, giving 364 responders and 362 non-responders in the reference set. The partition was selected by encouraging strong treatment separation, and as such is suitable as a target for future responder prediction. It also may be useful for hypothesis generation in testing for biomarkers of response with the caveat that strong results are expected and results should be replicated. The method includes all samples as labeled responders and non-responders, and uses similarity metrics when creating synthetic subgroups, which may yield more stable biomarkers. The Kaplan-Meier plot of the internal reference is shown in Fig. 5.

### Leave-one-out testing and predictive accuracy

**Fig. 6:**
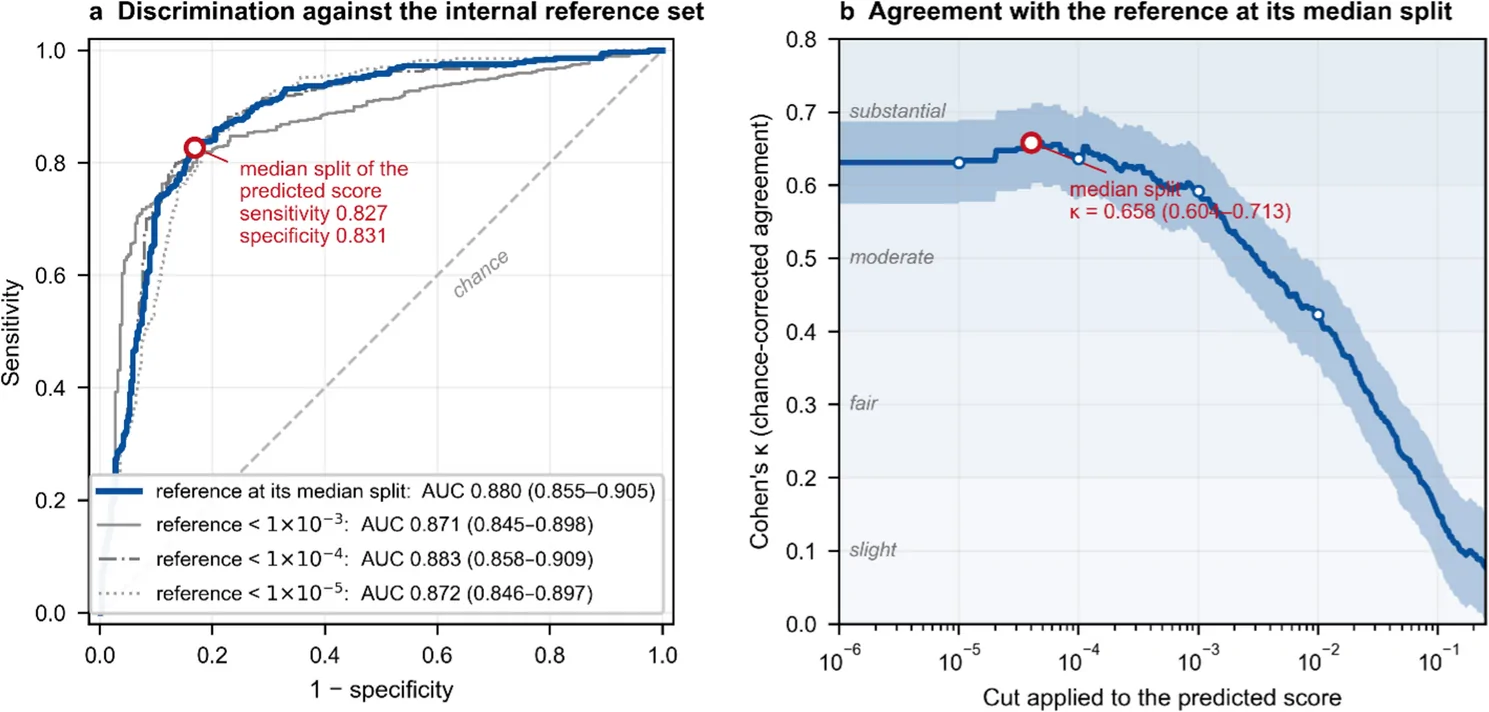
**a** AUC of sensitivity and specificity across p-value sweep for out-of-sample responder prediction, with multiple reference p-value thresholds visible and (**b**) agreement of response classification between predicted and internal reference, illustrated by Cohen’s к across p-value sweep.

The leave-one-out test procedure was conducted, and log-rank p-values of synthetic blocks using similarity across geometries of all remaining samples were used to test various cutpoints. Across the full range of cutpoints the leave-one-out score discriminated the internal reference labels with an area under the curve of 0.880 (95% CI 0.855–0.905) as shown in Fig. 6. At the median cutpoint of the predicted score, 5 × 10⁻⁵, sensitivity was 0.827 and specificity 0.831, with positive and negative predictive values of 0.831 and 0.827.

Table 2 gives these metrics across all cutpoints used. The prediction agreed with the internal reference labels for 82.9% of patients (Cohen’s κ = 0.658, 95% CI 0.604–0.713). The agreement was not strongly dependent on cutpoint, as accuracy stayed above 0.80 and κ above 0.60 from 10⁻⁶ to 8.6 × 10⁻⁴, and both reached their maximum at 5 × 10⁻⁵. The characteristics of patients classified as responders and non-responders via leave-one-out prediction at the median split are given in Table 3. Excluding the outcome PFS, age was the only significant variable, with predicted responders being 2.0 years younger on average (*p* = 0.009).

**Table 2:** Partitioning of patients and concordance, specificity and sensitivity rates using various p-value cutpoints during leave-one-out prediction procedure.

Cut on predicted score	Predicted responders *n* (%)	Operating characteristics					
		Sensitivity	Specificity	PPV	NPV	Accuracy	κ
5 × 10⁻²	627 (86.4)	0.978	0.251	0.568	0.919	0.616	0.230
1 × 10⁻²	553 (76.2)	0.973	0.450	0.640	0.942	0.712	0.423
1 × 10⁻³	462 (63.6)	0.931	0.660	0.734	0.905	0.796	0.592
1 × 10⁻⁴	380 (52.3)	0.841	0.796	0.805	0.832	0.818	0.636
5 × 10⁻⁵	362 (49.9)	0.827	0.831	0.831	0.827	0.829	0.658
1 × 10⁻⁵	308 (42.4)	0.739	0.892	0.873	0.773	0.815	0.631

Agreement is measured against the internal reference classification at its median split of 2 × 10⁻⁵ (364 responders, 362 non-responders)

**Table 3:** Patient characteristics of leave-one-out predicted responders and non-responders, at the median responder split.

Characteristic	Predicted responder (*n* = 362)	Predicted non-responder (*n* = 364)	*p*-value
Age, years - mean (SD)	59.7 (10.4)	61.7 (9.6)	0.009
Female - n (%)	90 (24.9%)	88 (24.2%)	0.830
PD-L1 positive - n (%)	260 (71.8%)	256 (70.3%)	0.657
TCGA cluster m3 - n (%)	124 (34.3%)	107 (29.5%)	0.160
PFS event observed - n (%)	196 (54.1%)	162 (44.5%)	0.009
Avelumab+Axitinib arm - n (%)	179 (49.4%)	175 (48.1%)	0.712

### Treatment effect heterogeneity

In the primary analysis, the predicted score entered as a continuous rank-transformed covariate, requiring no cutpoint. The treatment × predicted response interaction was significant in both Cox (*p* = 0.018) and RMST (*p* = 0.033) models. Dichotomizing at the median gave a ratio of hazard ratios of 0.53 (*p* = 0.0030) and an adjusted RMST of + 2.54 months in responders and + 0.44 months in non-responders, an interaction of + 2.10 months (*p* = 0.0044). The predicted responder group therefore accounted for 85.3% of the combined between-arm RMST gain (2.54 / [2.54 + 0.44]), against 50% expected in the absence of heterogeneity. Adjustment moved every interaction estimate further from the null, indicating that the heterogeneity is not explained by ​covariate imbalance. The full set of analyses across cutpoints is shown in Table 4.

**Table 4:** Treatment effect by predicted responder status across threshold cutpoints, Cox and restricted mean survival time models. The highlighted row is the median split.

Threshold	*n *resp / non	Cox interaction (ratio of hazard ratios)			ΔRMST₁₄ within each group, adjusted		RMST interaction
		unadjusted	+ age	+ age, sex, PD-L1	predicted responders	predicted non-responders	adjusted
5 × 10⁻²	627 / 99	0.793 *p* = 0.453	0.728 *p* = 0.307	0.730 *p* = 0.312	+ 1.59 (+ 0.80 to + 2.38) *p* = 8.1 × 10⁻⁵	+ 0.71 (− 1.20 to + 2.62) *p* = 0.468	+ 0.88 *p* = 0.402
1 × 10⁻²	553 / 173	0.807 *p* = 0.400	0.742 *p* = 0.243	0.734 *p* = 0.227	+ 1.65 (+ 0.80 to + 2.51) *p* = 1.4 × 10⁻⁴	+ 0.93 (− 0.48 to + 2.34) *p* = 0.198	+ 0.73 *p* = 0.388
1 × 10⁻³	462 / 264	0.628 *p* = 0.038	0.592 *p* = 0.020	0.590 *p* = 0.019	+ 2.07 (+ 1.13 to + 3.01) *p* = 1.6 × 10⁻⁵	+ 0.43 (− 0.71 to + 1.58) *p* = 0.461	+ 1.64 *p* = 0.030
1 × 10⁻⁴	380 / 346	0.607 *p* = 0.021	0.581 *p* = 0.012	0.579 *p* = 0.012	+ 2.35 (+ 1.33 to + 3.37) *p* = 5.9 × 10⁻⁶	+ 0.53 (− 0.51 to + 1.56) *p* = 0.317	+ 1.82 *p* = 0.014
**5 × 10⁻⁵**	**362 / 364**	**0.550** ***p*** **= 0.005**	**0.529** ***p*** **= 0.003**	**0.527** ***p*** **= 0.003**	**+ 2.54 (+ 1.50 to + 3.58)** ***p*** **= 1.6 × 10⁻⁶**	**+ 0.44 (− 0.57 to + 1.45)** ***p*** **= 0.394**	**+ 2.10** ***p*** **= 0.004**
1 × 10⁻⁵	308 / 418	0.654 *p* = 0.049	0.622 *p* = 0.028	0.622 *p* = 0.028	+ 2.33 (+ 1.20 to + 3.46) *p* = 5.3 × 10⁻⁵	+ 0.78 (− 0.16 to + 1.73) *p* = 0.104	+ 1.55 *p* = 0.040

### Gene results for internal classification and LOO prediction

Of 22,955 genes tested, 9,808 (42.7%) reached q < 0.05 against the internal reference score, which reflects the construction of a score built to maximize survival separation. The strongest associations were SOX13, HRC, PTPRB, EDNRB and LDB2 (all ρ ≈ −0.41; maximum |ρ| 0.41), and 99 of the top 100 genes were underexpressed in responders.

No individual gene is clearly identifiable as a proxy for the region. The leading genes each are co-expressed with up to 119 others above |r| = 0.7 and several hundred above 0.5. Retesting each gene after removing its co-expressed neighbors eliminated 67% of the transcriptome-wide association, leaving 3,238 genes and no top 10 gene was significant (smallest q = 0.079). Clustering resulted in 38 co-expressed clusters of five or more genes; conditioning on the first, a 476-gene cluster, removed 61% of the association, and subsequent conditioning on the second removed the remainder. These clusters explain 17.5% and 25.0% of the variance in the score, respectively, so they are not themselves strong predictors of the score.

The ranking is reproducible even though the cluster boundaries are not. Across 200 subsamples, ranking recovered 82% of the full-cohort top 100 (pairwise Jaccard 0.60), against 77% (0.53) for the same score dichotomized and 60% (0.29) for a median split of observed survival time. Cluster membership reproduced worse than the ranking itself (Jaccard 0.51–0.56 versus 0.69–0.72).

Out of fold, the best gene-based predictor reached an AUC of 0.758 for the internal reference classification and 0.738 for the leave-one-out prediction, in both cases using five cluster eigengenes (Table 5a). Reducing to two eigengenes, or expanding to the mean of the top 100 genes or to L2-penalized regression on all 22,955 genes, gave no improvement. Each of these reduced to between 0.699 and 0.755, against a baseline of 0.501. Gene-level information therefore appears to saturate at two to five co-expression modules, and a conventional classifier does not exceed that ceiling. The geometric score predicts the same internal reference classification at 0.880, recovering a further 0.12 beyond what expression alone provides.

**Table 5:** Co-expression gene modules and AUC measurements on internal reference and LOO prediction. (a) Out-of-fold AUC for each label, for the various predictors. (b) Treatment × module-score interaction on out-of-fold scores. (c) The 30-gene panel, split by direction of association with predicted response.

a. Out-of-fold AUC for predicting each geometric label		
Predictor	Internal reference	LOO prediction
Five cluster eigengenes	0.758	0.738
Two cluster eigengenes	0.755	0.699
Mean of the top 100 genes	0.740	0.722
Ridge logistic, all 22,955 genes	0.733	0.712
30-gene panel	—	0.721
Majority baseline	0.501	0.501

**b. Treatment × module-score interaction**,** out-of-fold scores**		
**Score**	**Ratio of hazard ratios**	**ΔRMST₁₄**,** responders vs. non (months)**
**LOO predicted score (primary analysis)**	**0.771 (** ***p*** ** = 0.018)**	**+ 2.42 vs. + 0.60**
Five modules, from the LOO label	0.652 (*p* = 0.0001)	+ 2.18 vs. + 0.79
Two modules, from the LOO label	0.780 (*p* = 0.021)	+ 1.93 vs. + 1.05
Five modules, from the reference label	0.654 (*p* = 0.0002)	+ 2.49 vs. + 0.36
Two modules, from the reference label	0.629 (*p* < 0.0001)	+ 2.53 vs. + 0.25

**c. The 30-gene panel**		
**Lower in predicted responders - endothelial and vascular**	**Higher in predicted responders - proliferation and cell cycle**	
PODXL, PTPRB, ESAM, LDB2, PLVAP, MMRN2, EDNRB, ATP1B2, A2M, MRVI1, SOX13, LDB3, ITGA8, GPR116, HRC	AURKA, BIRC5, CCNE1, CDK4, UBE2C, RRM2, CKS2, ORC6, NME1, TUBA1C, GARS, ALG3, PGAM5, TAF10, OAZ1	

The modules also reproduced the treatment effect separation of the full classifier. Out-of-fold scores from five modules derived from the leave-one-out label gave a ratio of hazard ratios of 0.652 (*p* = 0.0001), against 0.771 (*p* = 0.018) for the classifier itself. By the median split, the corresponding gains were + 2.18 versus + 0.79 months and + 2.42 versus + 0.60 months (Table 5b). Both labels identified the same dominant program, their leading cluster summaries correlating at 0.99 although gene membership overlapped only moderately (448 shared genes, Jaccard 0.43). Discrimination saturated at 20 genes (AUC 0.721) and did not improve beyond 50.

The frozen 30-gene panel separates by biology: 15 underexpressed in predicted responders are endothelial and vascular: PODXL, PTPRB, ESAM, LDB2, PLVAP, MMRN2, EDNRB, ATP1B2, A2M, MRVI1, SOX13, LDB3, ITGA8, GPR116, HRC. Fifteen overexpressed are proliferation and cell cycle: AURKA, BIRC5, CCNE1, CDK4, UBE2C, RRM2, CKS2, ORC6, NME1, TUBA1C, GARS, ALG3, PGAM5, TAF10, OAZ1 (Table 5c).

### Permutation results

**Fig. 7:**
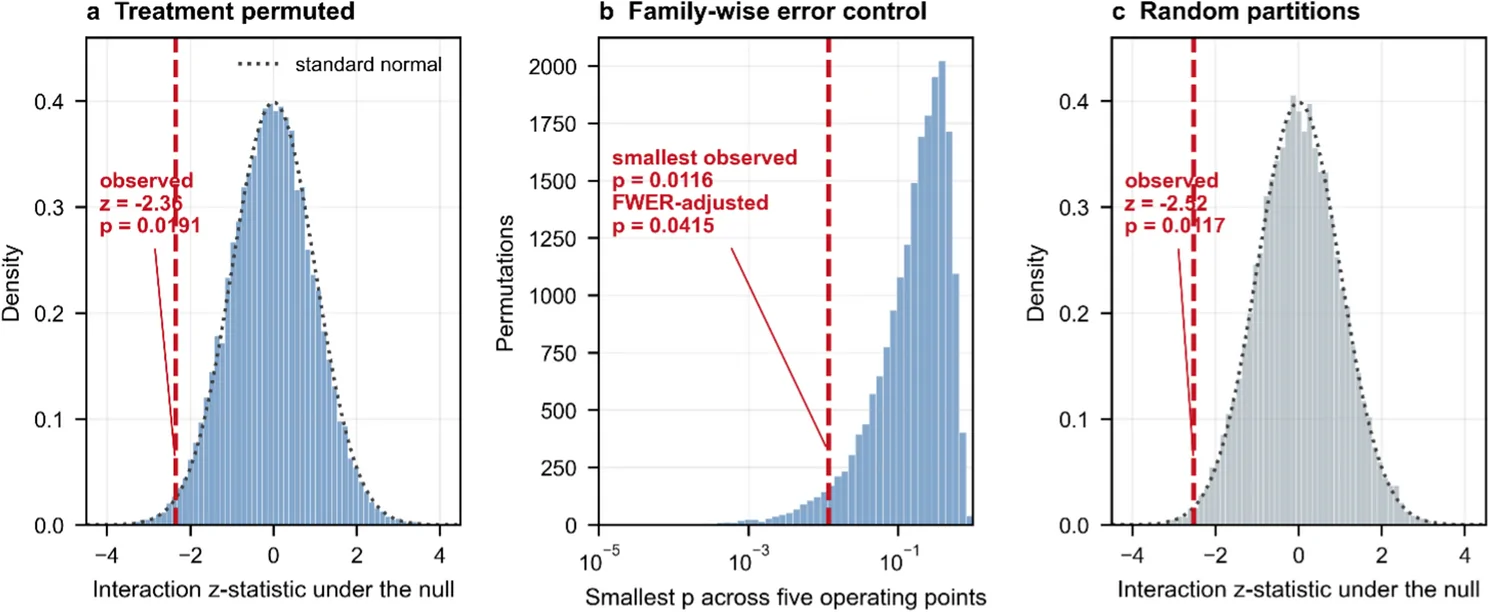
Permutation testing, 20,000 permutations. **a** Treatment permuted within PD-L1 × predicted responder strata; null matches the standard normal (dotted), observed z = − 2.36, *p* = 0.0191. **b** Smallest p across five operating points, Westfall–Young adjusted *p* = 0.0415. **c** Random partitions of equal size result in 1.2% as extreme as observed.

Treatment labels were permuted within PD-L1 × predicted responder strata across 20,000 permutations, with each original patient retaining their own survival time, censoring status, covariates and predicted score. The resulting null was centered at zero with unit variance (mean z = − 0.040, SD 1.006), and the permutation p-value for the primary test matched its asymptotic value nearly identically (0.019 versus 0.018), confirming that the Cox p-values are calibrated at this sample size (Fig. 7a).

The primary analysis treats the predicted score as continuous and requires no cutpoint. It gave a permutation p of 0.019 (Monte Carlo 95% CI 0.017–0.021) for the treatment × predicted response interaction (Table 6).

**Table 6:** Treatment effect heterogeneity permutation testing, using 20,000 permutations of treatment within PD-L1 × predicted responder strata, for the continuous score and at each operating point.

Analysis	Statistic	Asymptotic *p*	Permutation *p* (95% MC CI)	Adjusted *p*
**Primary analysis - predicted score as a continuous variable**				
**Treatment × predicted response interaction**	**z = − 2.364**	**0.0181**	**0.0191 (0.0172–0.0210)**	**NA**
**Secondary analysis - interaction at each operating point**				
Cut on predicted score 5 × 10⁻²	z = − 1.012	0.3117	0.3122	q = 0.312
Cut on predicted score 1 × 10⁻²	z = − 1.208	0.2272	0.2149	q = 0.284
Cut on predicted score 1 × 10⁻³	z = − 2.336	0.0195	0.0194	q = 0.047
Cut on predicted score 1 × 10⁻⁴	z = − 2.524	0.0116	0.0113	q = 0.047
Cut on predicted score 1 × 10⁻⁵	z = − 2.198	0.0280	0.0309	q = 0.047
**Multiplicity control across the five operating points**				
Westfall–Young min-p (family-wise error)	—	—	0.0416 (0.0388–0.0444)	—
Benjamini–Hochberg (false discovery rate)	—	—	—	q = 0.047
Bonferroni (reference only)	—	—	—	0.058
**Null distribution of the partition itself**				
Random size-matched partitions	z = − 2.524	—	0.0117 (0.0102–0.0132)	—

Across the five supplementary operating points, the permutation p ranged from 0.011 to 0.312. Controlling the family-wise error rate across them by the Westfall–Young min-p procedure gave an adjusted p of 0.042; Benjamini–Hochberg control gave q = 0.047 (Fig. 7b). Because the primary analysis is continuous without requiring a cutpoint, no adjustment applies to it.

To test the likelihood of a partition of this kind arising by chance, 20,000 random partitions matching the observed group sizes were generated and the interaction was recomputed in each. Of these, 1.2% produced an interaction as extreme as that observed (Fig. 7c).

### Comparator signature evaluations

Comparison with three published signatures is given in Table 7, each split at the median of its own score and evaluated in the same unadjusted models. The Renal 101 Immuno signature is reported under both mean and median aggregation. Our classification gave a treatment-by-subgroup interaction of 0.550 on the ratio of hazard ratios scale (95% CI 0.361 to 0.837, *p* = 0.005) and + 1.98 months on the RMST scale (95% CI + 0.52 to + 3.45, *p* = 0.008).

**Table 7:** Comparison with published gene-expression signatures, each split at the median of its own score and evaluated in the same unadjusted models, τ = 14 months. ΔRMST₁₄ is the between-arm difference within each predicted group. The Renal 101 Immuno signature was derived on this cohort.

Method	Genes	*n *resp/non	Cox interaction ratio of hazard ratios	RMST₁₄ interaction (months)	ΔRMST₁₄ within each group (months)	
			unadjusted	unadjusted	predicted responders	predicted non-responders
**Responder classification** **This cohort**,** out-of-sample**	**-**	**362/364**	**0.550 (0.361–0.837)** ***p*** **= 0.005**	**+ 1.98 (+ 0.52 to + 3.45)** ***p*** **= 0.008**	**+ 2.53** **(+ 1.48 to + 3.57)**	**+ 0.52** **(− 0.49 to + 1.54)**
Renal 101 Immuno, mean This cohort, in sample	26	363/363	0.703 (0.460–1.073) *p* = 0.102	+ 1.27 (− 0.20 to + 2.74) *p* = 0.090	+ 2.11 (+ 1.07 to + 3.15)	+ 0.94 (− 0.09 to + 1.98)
Renal 101 Immuno, median This cohort, in sample	26	363/363	0.668 (0.438–1.019) *p* = 0.061	+ 1.40 (− 0.07 to + 2.87) *p* = 0.062	+ 2.15 (+ 1.12 to + 3.18)	+ 0.84 (− 0.20 to + 1.89)
IMmotion150 Teff External	5	363/363	0.700 (0.460–1.065) *p* = 0.095	+ 1.32 (− 0.16 to + 2.79) *p* = 0.081	+ 2.12 (+ 1.08 to + 3.16)	+ 0.87 (− 0.18 to + 1.91)
Tumour inflammation signature External	18	363/363	0.823 (0.542–1.252) *p* = 0.364	+ 0.71 (− 0.77 to + 2.19) *p* = 0.349	+ 1.82 (+ 0.78 to + 2.86)	+ 1.18 (+ 0.13 to + 2.23)

No comparator reached significance on either scale, with ratios of hazard ratios from 0.668 to 0.823 (*p* = 0.061 to 0.364) and RMST interactions from + 0.71 to + 1.40 months (*p* = 0.062 to 0.349). The Renal 101 Immuno signature was derived on this cohort with these outcomes available during gene selection, and is therefore considered to be an in-sample evaluation. The IMmotion150 Teff and tumor inflammation signatures were derived in other cohorts and are considered to be external. Our reimplementation of the Renal 101 Immuno signature reproduced its published within-arm hazard ratios (0.62 versus 0.60 in the combination arm; 0.93 versus 0.89 in the control arm).

## Discussion

We present a method for using geometrical signatures based on tumor gene-expression in oncology clinical trials to identify the internal reference of responders as well as predict individual response with high accuracy. Our out-of-sample predictions stratify responders with 85.3% of the between-arm adjusted RMST gain in the predicted responder group. This use case shows strong utility for our method for cancer trial data and precision oncology.

These methods have key advantages over standard machine learning (ML) and artificial intelligence (AI) implementations applied directly to the data. Our method is effective with less than 1000 data points when the number of features is on the order of tens of thousands. Typically, ML and AI techniques require a large number of datapoints to train models, especially when noisy point clouds consist of huge numbers of variables. In contrast, our TDA method has denoising properties enabling identification of signals of otherwise non-observable geometrical patterns. The geometrical representation of the data via this method also inherently anonymizes data. For example, geometrically transformed gene-expression signatures cannot be reconverted to raw data by any means. This transformation can therefore be used without revealing patient data if it is sensitive in nature.

Our classification detected differential treatment benefit that established immune signatures did not, on the same patients and in the same models. This includes the Renal 101 Immuno signature, whose genes were selected based on differences in the progression-free survival outcomes of this cohort, while our score was predicted out-of-sample. Some of these signatures were derived on other platforms and in other tumor types, which may reduce their performance here. Nevertheless, reaching a significant result where an in-sample derived signature does not is a strong indication that the method is robust in detecting responders and their associated gene modules. Current methods in precision oncology typically prioritize mutations and addressable oncogenes, which derive from basic research and a need to identify the biological mechanisms underlying mode of action of a compound. However, as the DepMap project illustrated, gene-expression shows the strongest correlation with treatment response in cell-lines [6]. The use of the measurements at all genes leverages the power of geometry where complex​ relationships between variables may exist and are difficult to model, and where the use of some subset of variables is likely to reduce signal substantially. Because our geometrical signatures are built in a completely unsupervised fashion and without any input from treatment response, these transformations are robust and any correlation with treatment response is not built in via construction. Homological invariants are considerably less sensitive to the presence of noise and measurement precision, as well as the metric used or presence of normalization.

There are several benefits to utilizing this approach. First, it classifies all patients regardless of intervention or outcome with respect to the event or censoring. In the patients included in this study, only 157 patients (21.6%) were both treated and reached the PFS endpoint. Our method allows for full classification and removes any need for delicacy when a patient outcome or censoring might leave the response level in question. Second, the creation of biomarkers based on this approach may be more likely to be stable than doing a differential gene (DE) analysis. With over 20,000 genes routinely measured in RNA-seq, the variability in measurement tends to fill the expected distribution, so that any associations are likely to be spurious. Ranking genes against the geometric score recovered 82% of the top 100 genes across resampled subsets (pairwise Jaccard 0.60), against 60% (0.29) when the same genes were ranked against a median split of observed survival time. Third, as the adjusted RMST analysis in predicted responders/non-responders demonstrates, the geometry is linked with differential treatment benefit and not just gene expression structure. This also reinforces that the biomarkers detected are a result of geometry and thus might be hallmarks of response instead of simply being randomly distributed.

Indeed, in our gene-level test, the signal detected appeared as modules, without individual genes surviving conditioning on co-expressed genes in both our internal reference and LOO prediction dataset. Two to five modules were able to be selected using our geometrical setup conditions, which then exhaust transcriptomic information about response classification, likely to sufficient accuracy to have utility in either replication testing or future clinical tests. Against the internal reference classification, the LOO prediction score using geometrical methods reached an AUC of 0.880, while five eigenclusters achieved 0.738, suggesting the modules recover the majority of the discrimination. The module and gene results are cross-validated, with model fitting conducted inside each training fold. However, they still must be considered internal validation since the target label originates from these same 726 patients. Validation requires an independent cohort with the gene/module list and scoring procedures to be fixed in advance.

Patients predicted to benefit from adding avelumab to axitinib show lower endothelial and vascular expression and higher proliferative expression. The direction is highly coherent as tumors with high angiogenic output can be treated with a VEGFR alone, whereas tumors driven by proliferation rather than vascular dependency are most likely to gain from the addition of a checkpoint blockade. Our method was given no pathway annotation and recovered this contrast from expression and survival data alone.

These methods can be applied in Phase II trials to determine inclusion/exclusion criteria for Phase III studies, as well as to create additional primary or secondary endpoints for analysis. This might increase the chances of passing Phase III substantially. In failed Phase III trials, these techniques may be able to detect similar subgroups of responders not visible with the analytical methods used. This represents an enormous opportunity, since hundreds of oncology drugs have failed Phase III trials, the majority due to lack of efficacy. Identifying the most efficacious subgroups in a post-mortem fashion may allow for application of a fast-tracked trial to confirm the finding. Also, biomarkers from these analyses might be used to create companion diagnostics for responder detection in approved drugs.

## Conclusion

The application of geometrical gene-expression signatures identified both internal reference response classification as well as out-of-sample prediction of 82.9% accuracy in identifying responders at median response split in a cross-validated setting. Predicted responders accounted for 85.3% of the between-arm RMST gain after adjustment for age, sex and PD-L1 status. Established immune signatures evaluated on the same patients did not detect this heterogeneity. The method’s partitioning also detects between two and five modules of co-expressed genes which may identify responders sufficiently without requiring the full geometrical method to be present.

While this trial met primary endpoints, in Phase II/III studies the ability to detect and predict response subgroups may dramatically improve chances of approval, and these methods may be used for companion diagnostics or to identify target biomarkers of response for further testing.

## Data Availability

All data generated or analyzed during this study are included in this published article and its supplementary information files: https://pmc.ncbi.nlm.nih.gov/articles/PMC8493486/ The data are available in Supplementary tables: https://pmc.ncbi.nlm.nih.gov/articles/PMC8493486/#SD2 with direct download link at: https://pmc.ncbi.nlm.nih.gov/articles/instance/8493486/bin/NIHMS1737783-supplement-Supplementary_Tables.xlsb.

## Abbreviations

KM: Kaplan–Meier
AUC: Area under the receiver operating characteristic curve
CI: Confidence interval
RMST: Restricted mean survival time
FDA: Food and Drug Administration
DepMap: Dependency Map
RNA-seq: RNA sequencing
TDA: Topological data analysis
T2D: Type 2 diabetes
HLA: Human leukocyte antigen
PFS: Progression-free survival
TPM: Transcripts per million
PD-L1: Programmed death-ligand 1
HR: Hazard ratio
LOO: Leave-one-out
ROC: Receiver operating characteristic
Teff: T-effector
TCGA: The Cancer Genome Atlas
SD: Standard deviation
PPV: Positive predictive value
NPV: Negative predictive value
MC: Monte Carlo
ML: Machine learning
AI: Artificial intelligence
DE: Differential expression
VEGFR: Vascular endothelial growth factor receptor
